# Generative AI-related employment threat, AI optimism, and employment anxiety among domestic and international students at a Chinese university

**DOI:** 10.3389/fpubh.2026.1927670

**Published:** 2026-10-20

**Authors:** Yongming Huang, Yuyao Hu, Chunyu Ling, Xichen Liao, Xueyong Xiang, Qiujing Pan

**Affiliations:** Central South University, Changsha, China

**Keywords:** cognitive appraisal theory, cross-cultural comparison, employment anxiety, generative artificial intelligence, structural equation model

## Abstract

The rapid diffusion of generative artificial intelligence (AI) is reshaping the labor market, yet it remains unclear whether students’ appraisal of AI-driven employment threat translates into employment anxiety in similar ways across cultural groups. This study administered an online questionnaire to 1,595 domestic students (1,095) and international students (500) enrolled at Central South University. Perceived AI replacement threat, AI optimism, and employment anxiety were assessed with 13 five-point Likert items. Information channels, coping behaviors, desired institutional support, and perceived coping relief were assessed as supplementary measures. Confirmatory factor analyses supported an acceptable three-factor structure in both groups. The distinction between threat and anxiety remained qualified due to their substantial association. Metric invariance was established, and partial scalar invariance was obtained after the intercept of Q21 was freed. Perceived AI replacement threat and employment anxiety remained empirically distinguishable, although their latent correlation was stronger among international students than among domestic students. In the structural model, threat was strongly associated with anxiety (*β* = 0.764) and weakly associated with optimism (*β* = 0.133). After threat was included, optimism showed a small negative conditional association with anxiety (*β* = −0.152). The indirect association through optimism was negative but limited in magnitude, and equivalent cross-sectional specifications fit the data equally well. This pattern is best interpreted as an exploratory statistical finding compatible with suppression, rather than confirmed evidence of a directional psychological mechanism. Supplementary coping analyses were descriptive and exploratory. These findings provide qualified empirical support for a three-factor framework of students’ responses to AI-related employment uncertainty, and highlight differences in threat-anxiety coupling across student populations. The results carry implications for targeted student support services addressing AI-related career concerns.

## 1 Introduction

The accelerating deployment of generative artificial intelligence (AI) is restructuring the labor market and redefining the knowledge and skills students need to lead fulfilling lives and work efficiently (1–3). Studies have confirmed that regions with a high adoption rate of AI face a more notable drop in employment ratio and AI impacts especially on the early-career workforce, substantially intensifying the uncertainty and anxiety among students in universities (4). In China, where the graduating cohort has reached historic highs and competition is severe, employment-related psychological distress among university students is already recognized as a salient public-health concern (5–7). Chinese universities also host substantial populations of international students. If the organization or strength of these appraisal differs across groups, instruments and counseling approaches developed for one group may not transfer directly to the other (8–10).

Despite growing empirical interest in AI-related employment anxiety, most existing studies treat positive and negative orientations toward AI as opposite poles of a single dimension (11, 12). Furthermore, whether the appraisal of employment threat related to AI translates into employment anxiety through the same psychological structure across cultural groups remains underexplored (13–15). Less is known about whether the latent appraisal architecture maps onto observable coping behaviors in a coherent way (16). To address these gaps, this study focuses on three core appraisal constructs: perceived AI replacement threat, AI optimism, and employment anxiety. Three core constructs across both domestic and international students were modeled to capture potential group differences. Beyond invariance of the three-factor structure measured, the difference of coupling magnitude between threat and employment anxiety across two groups was also considered. The study is guided primarily by cognitive appraisal theory and conservation of resources (COR) theory (17, 18), which posits that anxiety follows primarily from threat rather than from the absence of positive evaluation. This study employs structural equation modeling (SEM) to examine the measurement structure and associations among perceived AI replacement threat, AI optimism, and employment anxiety. Measurement invariance is assessed across domestic and international students. The checklist-based coping analyses are supplementary and exploratory.

### 1.1 The cross-cultural comparability of AI-related concern

As a foundational framework in stress and emotion research, cognitive appraisal theory has been widely adopted to explore individuals’ psychological responses to AI technological advancements across multiple research contexts (17). Primary appraisal concerns the potential threat or benefit of an event, while subsequent appraisal involves the resources available for responding to it. By integrating cognitive appraisal theory with information quality theory, Nguyen et al. (19) proposed two core cognitive constructs: threat appraisal and coping appraisal, further verifying that threat appraisal positively predicts negative emotions such as anger and anxiety among users. Shi et al. (20) designed the I-PACE model to examine challenge and hindrance appraisals as key mediating mechanisms among university students. And they found that AI job replacement anxiety is associated with both challenge and hindrance appraisals. Similarly, Ge et al. (21) confirmed the double-edged sword effect of AI awareness on career decision-making difficulties based on the theory: threat appraisal of AI exacerbates students’ employment anxiety, while benefit appraisal alleviates their career decision-making dilemmas. Perceived AI replacement threat refers to an individual’s cognitive appraisal of whether AI endangers one’s occupational future through job displacement, skill obsolescence, or intensified competition (22, 23). It is a primary appraisal of implications for the self rather than a forecast about the labor market in general (17). AI optimism refers to a benefit-oriented appraisal of AI as a skill-building tool that can enhance one’s employability (24, 25). Employment anxiety refers to the emotional distress that accompanies the perception that one’s career prospects are threatened (5, 26). In this study, AI optimism denotes a situational AI-related adaptive appraisal involving perceived employment opportunities and perceived capability to learn or use AI for career adaptation, without measuring dispositional optimism.

Threat appraisal and employment anxiety are expected to be closely related because evaluations of possible employment harm may accompany emotional distress. Their empirical distinction may nevertheless be affected by item content. Statements about worry, career uncertainty, and low confidence can reflect both cognitive evaluation and emotion. Existing AI anxiety measures also include several related domains, indicating that the boundaries among AI-related threat, confidence, and anxiety may be difficult to define (11). Since wording concerning worry, career uncertainty, and confidence may partly overlap across appraisal and emotional content, the empirical boundary between threat and anxiety is evaluated rather than assumed. Cross-cultural validation research further suggests that language and item interpretation can influence factor structure across populations (10). Cultural context may also affect how individuals interpret uncertainty and translate appraisal into emotion (13, 27, 28). International students may consider language demands, visa arrangements and unfamiliar labor markets when evaluating employment uncertainty. These conditions are possible explanations rather than measured mechanisms.

Cross-group interpretation therefore requires evidence that the constructs are measured comparably. Measurement invariance testing can help distinguish substantive differences from variation in item functioning or response patterns (8, 29). Based on theoretical reasons, this study examines whether perceived AI replacement threat, AI optimism, and employment anxiety form a common three-factor structure among domestic and international students. It then evaluates measurement invariance and compares the strength of the threat-anxiety association across groups. The study compares domestic and international students studying at the same Chinese university. It examines whether a common three-factor configuration is reasonably comparable across these student populations and whether the association between threat and anxiety differs in strength, without directly testing cultural mechanisms or compare national cultural systems.

### 1.2 The relationships among cognitive constructs

Cognitive appraisal theory holds that anxiety arises primarily from threat appraisal, instead of the absence of positive evaluation (20). A growing body of empirical research supports this prediction. Perceived AI threat and heightened AI awareness have been linked to job insecurity, emotional exhaustion, and employment anxiety in both workers and university students (7, 30). At the same time, the double evaluation logic of cognitive appraisal theory implies that optimism, as a benefit-oriented appraisal, operates through a channel separate from the pathway linked threat and anxiety (31). Recent studies have documented that people can simultaneously endorse strong AI-related concerns and positive attitudes toward AI (32). Therefore, optimism was examined as a separate construct whose association with anxiety may depend on perceived threat.

COR theory offers one possible interpretation: concerns about employment resources may coexist with interest in acquiring new skills (18, 33). A structural model linking threat, optimism, and anxiety, including an indirect association through optimism, was estimated. Cross-sectional data also permit alternative directional and common-cause explanations (34). A separate analysis tests whether optimism moderates the association between threat and anxiety.

In this study, SEM estimates a cross-sectional indirect association through AI optimism. COR theory provides one possible interpretation, but reversed, reciprocal, and common-cause explanations are also compatible with the design. The indirect pattern and the moderation analysis are therefore treated as exploratory statistical analyses rather than as tests of a temporal mechanism. They may cancel at the bivariate level, so that the statistical pattern is called as a suppression effect (35–37). If positive and negative appraisals operate through independent channels, optimism should not weaken the strength of the link between threat and anxiety. This study examines whether the associations among threat, optimism and anxiety is consistent with such a suppression configuration. Due to the cross-sectional data, this configuration is treated as one of several statistically equivalent representations of the associations.

### 1.3 From constructs to coping behavior

The third theoretical question focuses on how these psychological patterns are associated with self-reported coping behavior. In cognitive appraisal theory, primary appraisal is followed by secondary appraisal, in which individuals evaluate their coping resources and select coping strategies (38, 39). The emotion regulation model further distinguishes adaptive coping strategies, such as cognitive reappraisal, from maladaptive strategies, such as avoidance (40–42). This study includes a supplementary exploratory checklist of coping behaviors, information channels, and support preferences. The analyses describe their cross-sectional associations with the three core constructs and a single-item report of overall perceived relief, without establishing causal links between specific coping behaviors and changes in anxiety or relief. Figure 1 illustrates the conceptual model depicting the hypothesized relationships among these variables across the three theoretical stages of construct establishment, construct relationships, and behavioral mapping.

**Figure 1:**
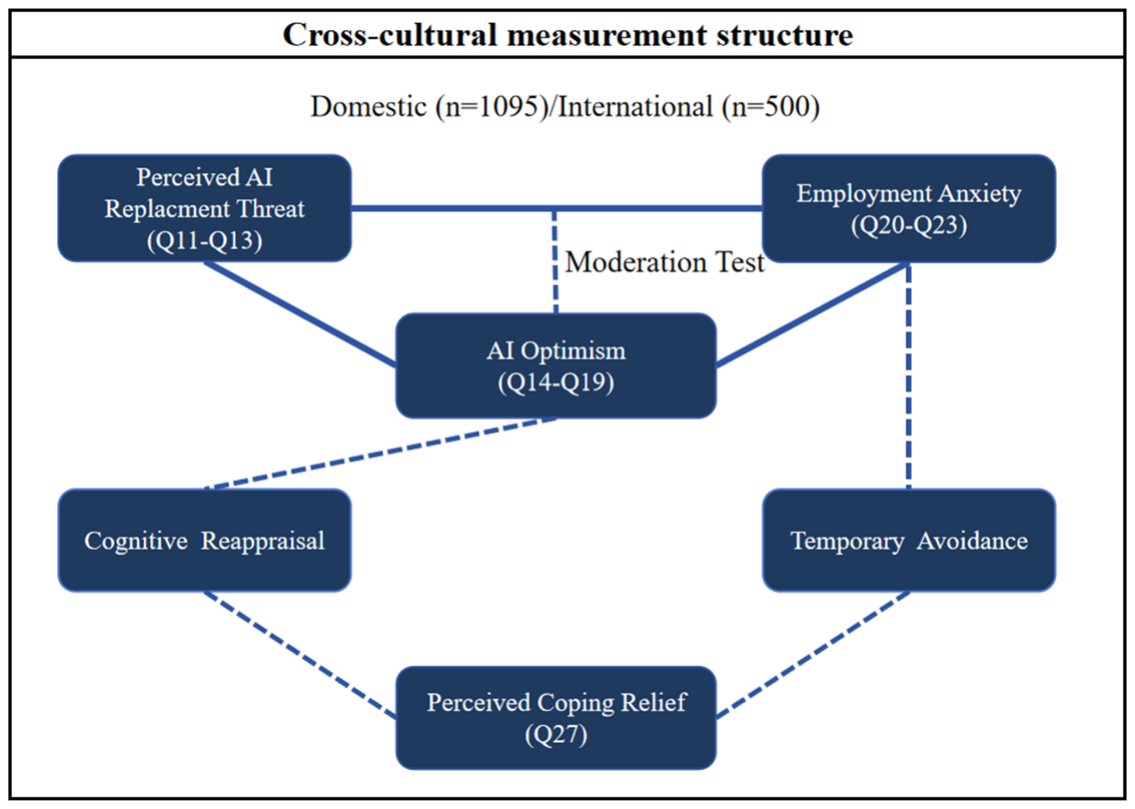
Conceptual framework in this study.

## 2 Methods

### 2.1 Participants and procedures

A cross-sectional online questionnaire was administered to enrolled students at Central South University, Changsha, China, spanning from April to July 2026. Recruitment relied on convenience sampling through class groups and the international student office. All participants have covered all grades from freshman to doctoral students, and five major disciplinary categories, including Science, Technology, Engineering and Mathematics (STEM), Humanities and Social Sciences (HSS), Economics, Management and Law (EML), Arts and Physical Education, medicine, and others. They have provided informed consent and voluntarily agreed to participate. The inclusion criteria for participants were: (1) ability to understand and cooperate with the survey; (2) voluntary participation in the study. Exclusion criteria included: (1) incomplete questionnaire responses (less than 80% completion); (2) refusal to participate in the survey. After excluding missing values and invalid responses, 1,595 valid questionnaires were retained for analysis, comprising 1,095 Chinese domestic students and 500 international students. As shown in Table 1, demographic characteristics by group are reported. Males accounted for 58% of the sample. Around 70 percentage of international students have been in China for more than 1 year. Students with internship or part-time work experience are roughly equal to students without such experience. The only negative skewness value falls to AI familiarity among all variables, exhibiting a slightly left-skewed characteristic. 75.17% of students frequently use AI, and 72.60% of students face moderate financial stress.

**Table 1:** Descriptive statistics of demographic characteristics.

Variables	Dummy variable		Observation	Proportion	Mean	Variance	Skewness	Kurtosis
Gender	Male = 1		925	58.00%	1.42	0.24	0.32	−1.90
	Female = 2		670	42.00%				
Country	Domestic = 1		1,095	68.65%	1.31	0.22	0.81	−1.35
	International = 2		500	31.35%				
	Duration in China (international students)	< 1 year = 1	144	28.80%	2.16	1.31	1.55	1.25
		1–3 years = 2	199	39.80%				
		3–5 years = 3	90	18.00%				
		> 5 years = 4	67	13.40%				
Grade	Freshman = 1		509	31.91%	3.23	3.55	0.07	−1.55
	Sophomore = 2		155	9.72%				
	Junior = 3		198	12.41%				
	Senior = 4		123	7.71%				
	Master = 5		420	26.33%				
	Doctor = 6		190	11.91%				
Major disciplinary categories	STEM = 1		823	51.60%	2.36	3.00	0.95	−0.56
	HSS = 2		192	12.04%				
	EML = 3		204	12.79%				
	Arts and PE = 4		67	4.20%				
	Medicine = 5		180	11.29%				
	Other = 6		129	8.09%				
Part-time work experience	Yes = 1		800	50.16%	1.50	0.25	0.01	−2.00
	No = 2		795	49.84%				
Family financial pressure	Very low = 1		160	10.03%	2.09	0.33	0.67	1.87
	Moderate = 2		1,158	72.60%				
	Relatively high = 3		244	15.30%				
	Very high = 4		33	2.07%				
AI familiarity	Very familiar = 1		177	11.10%	2.47	0.33	−0.16	−0.22
	Relatively familiar = 2		585	36.68%				
	Moderately familiar = 3		737	46.21%				
	Relatively unfamiliar = 4		91	5.71%				
	Very unfamiliar = 5		5	0.31%				
AI using frequency	Frequently use = 1		1,199	75.17%	1.27	0.25	1.79	3.28
	Occasionally use = 2		364	22.82%				
	Heard but never used = 3		27	1.69%				
	Never heard = 4		5	0.31%				

In particular, the international student sample comprised 78 countries or territories as shown in Supplementary Table S1. Participants reported origins in Asia (*n* = 337, 67.4%), Africa (*n* = 124, 24.8%), Europe (*n* = 22, 4.4%), South America (*n* = 7, 1.4%), North America (*n* = 7, 1.4%), and Oceania (*n* = 3, 0.6%). The largest national groups were from Pakistan (*n* = 89, 17.8%), Turkmenistan (*n* = 40, 8.0%), Cambodia (*n* = 38, 7.6%), and Indonesia (*n* = 37, 7.4%). Among international students, 144 (28.8%) had been in China for less than 1 year, 199 (39.8%) for 1–3 years, 90 (18.0%) for 3–5 years, and 67 (13.4%) for more than 5 years. These characteristics describe the heterogeneity of the international students.

### 2.2 Measuring tools

Questionnaire development drew on researches on AI anxiety and attitudes toward AI (43–45). Based on the Artificial Intelligence Anxiety Scale (3) and the General Attitudes towards Artificial Intelligence (GAAIS) (46), the questionnaire initially established four candidate constructs related to AI and career perceptions: (1) perceptions of AI replacement threat, (2) attitudes toward AI, (3) confidence in AI usage, and (4) employment anxiety. The cross-cultural adaptation underwent forward and back translation (47) Two bilingual international students contributed translations and feedback, and two professors with overseas academic experience reviewed both versions. Discrepancies were resolved through discussion.

To verify the feasibility of developed questionnaires, a pilot study has been conducted with 326 students, comprising 187 domestic and 139 international students. Exploratory factor analysis (EFA) with principal component analysis and varimax rotation are performed on the collected data, showing a Kaiser-Meyer-Olkin (KMO) value of 0.862 and a significant Bartlett’s Test of Sphericity (*p* < 0.001). However, factor loading revealed a significant cross-loading issue: all six items originally designed to measure “attitudes toward AI” (Q14-Q17) and “confidence in AI usage” (Q18-Q19) loaded highly on a single common factor (ranging from 0.621 to 0.814), and factor loadings on their intended separate factors were all below 0.40.

The six items were retained as one empirically derived factor, termed AI optimism in this study. The label refers to a situational AI-related adaptive appraisal that combines perceived opportunity and perceived adaptive capability. After using factor analysis, its shared loading pattern provides evidence of internal structure, but it does not by itself establish that opportunity appraisal, learning orientation, and AI-related self-efficacy are interchangeable. The construct is therefore interpreted within this study-specific scope.

The final model contained 13 five-point Likert items: perceived AI replacement threat (Q11–Q13), AI optimism (Q14–Q19), and employment anxiety (Q20–Q23). Q11 includes affective wording, Q20 refers directly to AI replacement, and Q21 concerns general graduate employment. These features are considered in the discriminant-validity and invariance analyses. Besides, Q10 assessed expected job replacement across the labor market and was excluded to focus the threat measure on personal vulnerability. Q24 assessed the tone of encountered AI-related employment information and was excluded from the anxiety indicators. Descriptive statistics used to assess the three core constructs are presented in Table 2. The full questionnaire is described in Supplementary material.

**Table 2:** Descriptive statistics of the five-point Likert items (Q10–Q23).

Item	Content	Construct	Mean	Variance	Skewness	Kurtosis
Q10	AI will replace many existing jobs in the next 5–10 years	Excluded	3.55	0.91	−0.40	−0.08
Q11	I worry that my major is easily replaceable by AI	Threat	2.98	1.13	0.05	−0.56
Q12	AI will make me face fiercer competition in job-seeking	Threat	3.52	0.95	−0.47	−0.03
Q13	Even with effort, AI may render my skills obsolete	Threat	3.26	1.07	−0.23	−0.36
Q14	AI is a tool I can use, not a threat	Optimism	3.81	0.72	−0.51	0.44
Q15	I am willing to actively learn AI tools	Optimism	4.05	0.67	−0.84	1.25
Q16	Those who master AI have advantages in the job market	Optimism	4.06	0.70	−0.86	1.15
Q17	AI development will create more new job opportunities	Optimism	3.76	0.86	−0.50	0.13
Q18	I am confident I can learn to use common AI tools	Optimism	3.94	0.63	−0.57	0.68
Q19	Through learning AI, I can enhance my career competitiveness	Optimism	3.97	0.64	−0.65	0.94
Q20	Thinking that AI may replace my job makes me anxious	Anxiety	3.05	0.99	−0.06	−0.32
Q21	I worry about not finding a satisfactory job after graduation	Anxiety	3.38	1.18	−0.35	−0.49
Q22	AI development makes me feel lost about my career path	Anxiety	3.00	1.02	0.01	−0.35
Q23	I lack confidence in my competitiveness in the AI era	Anxiety	3.00	1.03	−0.02	−0.35

Additionally, the questionnaire comprised four supplementary modules: information channels (Q25), coping behaviors (Q26), self-reported perceived relief after coping (Q27), and desired institutional support (Q28). Coping options drew on appraisal and emotion-regulation research (17, 48), with AI learning and internship experience added for the employment context (49, 50). Q27 was a study-specific single item assessing perceived relief after coping without formal content or psychometric validation. Q28 was assessed with a multiple-response preference checklist of six options. The options, were generated on the basis of the graduate employ-ability and AI-literacy provision in higher education (51–53). These represented separate behaviors or preferences and were analyzed individually. In the study, Q25–Q28 were supplementary exploratory measures. The main analysis and conclusions rest on the three core constructs.

### 2.3 Statistical analysis

This study adopts SPSS R27.0.1.0, the PROCESS v5.0 macro, and R (v.4.5.0) with lavaan (v.0.7–2) and semTools (v.0.5–9) for all statistical analyses. EFA used principal-axis factoring with Promax rotation, with parallel analysis used to determine the number of factors. Confirmatory factor analyses were estimated with robust maximum likelihood (MLR), using robust standard errors and a scaled test statistic. Measurement invariance was examined sequentially at the configural, metric, and scalar levels. When full scalar invariance was not supported, score tests were used to identify non-invariant intercepts and estimate a partial scalar model (54). Latent means were compared under the partial scalar model, with the domestic subsample as the reference group and standardized against its latent standard deviation (55).

The structural model included perceived AI replacement threat, AI optimism, and employment anxiety as latent variables. Bias-corrected bootstrap 95% confidence intervals were estimated from 5,000 resamples. AI familiarity and usage frequency were included as covariates in a supplementary analysis, and equivalent structural specifications were examined as an additional robustness check (56). The interaction between threat and optimism was examined in a supplementary moderation analysis. Each checklist option of Q25–Q28 was exploratorily analyzed separately. Sensitivity analyses assessed the retention of Q10, the exclusion of items with potentially overlapping wording, the Q18–Q19 residual covariance on model fit and the primary structural estimates, and weighted least squares mean and variance adjusted (WLSMV) estimation for ordinal indicators, as reported in Supplementary Table S3.

## 3 Results

### 3.1 Measurement model

Parallel analysis suggested retaining three factors in each group. Principal-axis factoring with Promax rotation was therefore conducted separately within each group. Within the domestic sample, Q11-Q13 loaded on the threat factor (0.720–0.764), Q14–Q19 on the optimism factor (0.689–0.884), and Q20–Q23 on the anxiety factor (0.691–0.894). The three factors accounted for 62.3% of the item variance. The similar pattern was recovered in the international sample: Q12 and Q13 loaded on the threat factor, Q14–Q19 on optimism, and Q20–Q23 on anxiety. Q11 showed a primary loading of 0.543 on threat and a secondary loading of 0.369 on anxiety, representing the only notable cross-loading. The three factors accounted for 58.6% of the item variance in the international sample. The complete EFA pattern matrices are reported in Supplementary Table S2.

CFA was subsequently conducted on the final 13-item three-factor model in the full sample. The model showed an acceptable fit to the data, χ^2^(61) = 368.61, *p* < 0.001, Comparative Fit Index (CFI) = 0.966, Tucker-Lewis Index (TLI) = 0.957, Root Mean Square Error of Approximation (RMSEA) = 0.066, 90% CI (0.059, 0.072), and Standardized Root Mean Square Residual (SRMR) = 0.064. All standardized factor loadings were substantial: 0.737–0.748 for perceived AI replacement threat, 0.657–0.894 for AI optimism, and 0.759–0.890 for employment anxiety. These indices were consistent with the proposed representation, while leaving some residual misfit and item-level ambiguity. After inspection of modification indices, the Q18–Q19 residual covariance was added as a *post hoc* adjustment. Considering both items concern confidence in learning or using AI for employment-related advantage, their residual covariance may reflect item redundancy, local dependence, or a narrower facet that is not fully captured by the broader AI optimism factor. The present data cannot distinguish these explanations. As a sensitivity check, the same model without the Q18–Q19 residual covariance showed weaker fit (CFI = 0.943, TLI = 0.929, RMSEA = 0.084, SRMR = 0.066). The main standardized structural estimates changed little, including the indirect association, *β* = −0.021, compared with *β* = −0.020 in the primary model as shown in Supplementary Table S3.

As shown in Table 3, values below the diagonal are latent correlations, values above the diagonal are heterotrait monotrait ratio (HTMT) ratios. The three constructs also demonstrated satisfactory internal consistency and convergent validity. HTMT values were below the commonly used 0.85 criterion, but threat and anxiety remained strongly associated. The latent correlation for threat and anxiety (0.744) was approximately equal to the square root of the average variance extracted (√AVE) for threat (0.744), making the Fornell-Larcker result borderline rather than clearly satisfied. Composite reliability (CR) ranged from 0.788 to 0.900 and AVE from 0.553 to 0.692.

**Table 3:** Convergent and discriminant validity of the three core constructs.

Construct	Perceived AI replacement threat	AI optimism	Employment anxiety	CR	AVE
Perceived AI replacement threat	0.744	0.094	0.752	0.788	0.553
AI optimism	0.133	0.774	0.064	0.898	0.599
Employment anxiety	0.744	−0.051	0.832	0.900	0.692

The same CFA structure showed acceptable fit in both groups. Within the international sample, the same three-factor model showed χ^2^(61) = 162.34, CFI = 0.969, TLI = 0.961, RMSEA = 0.062, SRMR = 0.063. In the domestic sample, it showed acceptable fit, χ^2^(61) = 335.29, CFI = 0.954, TLI = 0.941, RMSEA = 0.077, SRMR = 0.074. Threat and anxiety remained separately specified constructs in both groups, although their empirical distinction was more limited in the international student sample. HTMT was 0.707 in the domestic sample and 0.813 in the international sample, both below the 0.85 threshold. The latent correlation between threat and anxiety was 0.70 among domestic students and 0.82 among international students. Supplementary Table S3d reports HTMT2 bootstrap intervals of (0.641, 0.769) for domestic students and (0.750, 0.869) for international students. The latter interval extends above 0.85 and therefore qualifies the evidence for discriminant validity among international students, consistent with the pattern of latent correlation estimates.

The first unrotated factor accounted for 29.91% of item variance. A single-factor CFA fitted poorly, with robust CFI = 0.460, TLI = 0.352, RMSEA = 0.255, 90% CI (0.248, 0.261), and SRMR = 0.258. These diagnostics indicate that a single common factor does not reproduce the covariance structure, but they do not rule out common method bias. As a supplementary measurement check, a two-factor model merging threat and anxiety was also estimated, fitting poorly in both groups as reported in Supplementary Table S2b.

### 3.2 Measurement invariance and group differences

Multi-group CFA was used to evaluate measurement comparability across groups as shown in Table 4. Configural invariance held with CFI = 0.956, RMSEA = 0.075, SRMR = 0.066. Adding equality constraints on factor loadings did not appreciably worsen fit (metric model: CFI = 0.954, RMSEA = 0.074, SRMR = 0.069; ΔCFI = −0.002, ΔRMSEA = −0.001, ΔSRMR = 0.003), meeting criteria for metric invariance. Especially, Q21 (“I worry about not finding a satisfactory job after graduation”) asks about graduate employment in general rather than about AI. The score test identified Q21 as the only substantially non-invariant intercept [χ^2^(1) = 117.72, next largest = 20.86]. Freeing this single intercept yielded partial scalar invariance (CFI = 0.953, RMSEA = 0.073, SRMR = 0.069), with ΔCFI = −0.001 relative to the metric model, a proportion well within accepted limits, Partial scalar invariance provides a limited basis for group comparisons. It does not establish complete measurement equivalence.

**Table 4:** Measurement invariance of the three-factor model.

Model	χ^2^ (df)	CFI	TLI	RMSEA	SRMR	ΔCFI
Configural	675.932 (122)	0.956	0.943	0.075	0.066	–
Metric	709.202 (132)	0.954	0.945	0.074	0.069	−0.002
Scalar	856.842 (142)	0.943	0.937	0.079	0.072	−0.011
Partial scalar	734.955 (141)	0.953	0.947	0.073	0.069	−0.001

The strength of the association between perceived AI replacement threat and employment anxiety differed across groups. Under the partial scalar invariance model, the latent correlation between threat and anxiety was 0.698, 95% CI (0.630, 0.759), in the domestic sample and 0.819, 95% CI (0.760, 0.877), in the international sample. The difference between the two correlations was statistically significant [Δ*r* = 0.121, 95% CI (0.036, 0.213), *p* = 0.007]. This estimate describes stronger threat-anxiety coupling among international students. It does not identify whether cultural context, language interpretation, employment destination, or another unmeasured factor produced the difference.

Latent means were subsequently compared under the partial scalar invariance model, with the domestic sample serving as the reference group (Table 5). International students showed lower latent levels of perceived AI replacement threat [ΔMean = −0.241, 95% CI (−0.341, −0.144), *p* < 0.001; standardized difference = −0.294] and employment anxiety [ΔMean = −0.358, 95% CI (−0.448, −0.258), *p* < 0.001; standardized difference = −0.414]. The latent mean difference in AI optimism was small and not statistically significant [ΔMean = 0.051, 95% CI (−0.017, 0.120), *p* = 0.140; standardized difference = 0.081]. Given partial scalar invariance and heterogeneity within the international sample, these latent-mean estimates should be interpreted cautiously. As a sensitivity check, the corresponding anxiety difference under the full scalar specification was −0.423, which was consistent in direction and statistical significance with the partial scalar result. Thus, freeing the intercept of Q21 did not alter the substantive conclusion regarding the group difference in employment anxiety.

**Table 5:** Structural model estimates with bias-corrected bootstrap 95% confidence intervals.

Path	Pathway	Unstandardized coefficient	Standardized coefficient	SE	*p*	95% bootstrap CI
Path a	Threat → Optimism	0.099	0.133	0.032	0.002	(0.036, 0.164)
Path b	Optimism → Anxiety	−0.205	−0.152	0.033	<0.001	(−0.266, −0.139)
Direct effect (c’)	Threat → Anxiety	0.765	0.764	0.037	<0.001	(0.694, 0.837)
Indirect effect (a × b)	Threat → Optimism → Anxiety	−0.020	−0.020	0.008	0.012	(−0.039, −0.007)
Total effect		0.745	0.744	0.035	<0.001	(0.678,0.814)

### 3.3 Mediation and moderation

As shown in Table 5, the structural equation model estimated that perceived AI replacement threat was strongly and positively associated with employment anxiety [path c′ = 0.765, *β* = 0.764, *p* < 0.001; 95% CI (0.694, 0.837)], Threat was also positively associated with optimism [path *a* = 0.099, *β* = 0.133, *p* = 0.002; 95% CI (0.036, 0.164)]. Controlling for threat, optimism was negatively associated with anxiety [path *b*: −0.205, *β* = −0.152, *p* < 0.001; 95% CI (−0.266, −0.139)]. The indirect effect through optimism was minimal and negative [path a × b = −0.020, 95% CI (−0.039, −0.007), *p* = 0.012; standardized indirect effect = −0.020], whereas the total effect of threat on anxiety was positive [total effect = 0.745, *p* < 0.001; 95% CI (0.678, 0.814)]. The model accounted for approximately 57.6% of the variance in employment anxiety.

The set of cross-sectional associations was statistically compatible with suppression. Equivalent specifications that reversed the threat-optimism path or represented it as a covariance showed identical fit. Supplementary covariate, observed-score, and moderation analyses are reported in Supplementary Tables S5–S7, consistent with the primary cross-sectional interpretation. The result is therefore treated as an exploratory statistical pattern rather than as evidence of temporal ordering or a psychological mechanism.

Additionally, the checklist findings were exploratory because information channels (Q25), coping behaviors (Q26), desired support (Q28), and self-reported perceived relief after coping (Q27) were assessed with study-specific items. Active AI skill learning and cognitive reappraisal were the most frequently endorsed coping options. Temporary avoidance co-occurred with higher employment anxiety and lower self-reported perceived relief, whereas cognitive reappraisal co-occurred with higher AI optimism and higher reported relief. These associations do not identify the direction of influence or the effect of an individual coping strategy.

## 4 Discussion

This study used structural equation modeling as the core analytic framework, encompassing EFA, CFA, multi-group measurement invariance testing, structural path estimation with bootstrapped mediation, and moderation analysis. It frames the research pathway from the AI stressor and primary appraisals (threat, optimism, and anxiety) to emotional responses and further to coping behaviors and perceived coping relief.

### 4.1 Appraisal architecture

The measurement results supported the same three-construct organization of perceived AI replacement threat, AI optimism, and employment anxiety among domestic and international students. This common structure is consistent with the distinction in cognitive appraisal theory between evaluating a potential threat and experiencing the emotional response associated with it (17). Threat and anxiety were closely related in both groups. The results supported retaining them as separate dimensions in the present model, yet the distinction remained qualified due to their substantial association, the borderline Fornell-Larcker result, and overlapping item content.

The stronger association between threat and anxiety observed among international students reflects a difference in how tightly these experiences are connected. Several factors may contribute to this difference. Language and item interpretation may influence how closely students distinguish statements about employment threat from statements about anxiety. The international sample was also heterogeneous in national and educational background, which may have introduced additional variation in how the items were understood (29). However, this study did not directly measure these mechanisms.

AI optimism, comprising learning orientation and career confidence, is interpreted here as a study-specific adaptive appraisal of AI-related opportunity and capability. Its reliability and convergent-validity estimates were adequate, but its broader construct validity requires further examination. It showed only a weak positive association with threat (*r* = 0.133) and a near-zero association with employment anxiety (*r* = −0.051). This pattern is consistent with previous findings that positive attitudes toward AI can coexist with AI-related concerns (32, 57). Latent-mean comparisons further showed that domestic students reported higher levels of perceived threat and employment anxiety than international students, whereas AI optimism did not differ significantly between the two groups.

### 4.2 Optimism to threat-anxiety association

Perceived AI replacement threat was the construct most closely associated with employment anxiety. This relationship is consistent with earlier research linking AI-related career concerns to anxiety about future employment (58, 59). AI optimism showed a more complex relationship. Its bivariate association with anxiety was minimal, yet a small negative association emerged after threat was taken into account. The resulting pattern is consistent with statistical suppression (34). This finding offers a useful way to understand why optimism and anxiety can coexist. Positive expectations about AI do not necessarily imply that students perceive less employment risk. A student may believe that AI creates opportunities for learning and career development while also worrying about displacement, competition, or changing skill requirements. Positive and negative evaluations can therefore remain psychologically relevant at the same time.

COR theory provides one possible interpretation. Employment threat may increase concern about future resources while also encouraging efforts to strengthen skills and adaptability (18). Students who engage more with AI may likewise become more aware of its risks while gaining confidence in their ability to use it. The covariate analysis indicates that familiarity and usage account for only part of this relationship. The direction of the relationship between threat and optimism remains uncertain. Models that specified different directional relations between the two constructs fit the data equally well, as reported in Supplementary Table S5 (56). The present data therefore support the coexistence of these appraisals more clearly than any particular temporal sequence between them. From a theoretical standpoint, COR theory is one possible interpretation of this pattern. The small indirect association and the identical fit of equivalent specifications prevent a directional interpretation.

Additionally, optimism also showed a statistically detectable but small moderating association with the link between threat and anxiety. Students with higher optimism tended to show a slightly weaker association between the two, although the relationship remained substantial across levels of optimism as shown in Supplementary Table S7.

The coping analyses were exploratory. Avoidance and cognitive reappraisal co-occurred with different psychological measures, but the study cannot determine whether coping preceded these measures or resulted from them. The group differences in checklist endorsement may reflect information access, academic context, social resources, or other unmeasured conditions rather than stable cultural coping styles.

## 5 Conclusion

Against the backdrop of generative AI reshaping employment landscapes and amplifying career uncertainty among university students worldwide, it remains an open question whether the psychological structure of AI-related employment appraisal operates consistently across diverse student groups. This study examined perceived AI replacement threat, AI optimism, and employment anxiety among domestic and international students at a comprehensive Chinese university, to test the generalizability of a three-construct appraisal framework and explore group differences in structural associations. A cross-sectional online questionnaire was administered to 1,595 participants (1,095 domestic students and 500 international students) at Central South University. Confirmatory factor analysis, multi-group measurement invariance testing, and structural equation modeling with bias-corrected bootstrap estimation were adopted to examine the measurement structure and inter-construct relationships. Supplementary exploratory analyses of self-reported coping behaviors and perceived coping relief were also conducted.

The core findings can be summarized as follows. First, a shared three-factor structure was supported across both student populations, and the final 13-item model demonstrated acceptable fit to the data. Perceived AI replacement threat and employment anxiety were distinguishable as separate constructs, but their substantial association and borderline Fornell-Larcker results indicated that their empirical distinction should be interpreted cautiously; the strength of the association between threat and anxiety was significantly greater among international students than among domestic students. Second, partial scalar measurement invariance was established, providing a valid basis for cross-group latent mean and correlation comparisons. Third, perceived AI replacement threat showed the strongest cross-sectional association with employment anxiety. After controlling for threat, AI optimism exhibited a small negative conditional association with anxiety, and the indirect association through optimism was limited in magnitude. Given that equivalent cross-sectional model specifications fit the data equally well, this pattern is treated as an exploratory statistical finding rather than confirmed evidence of a directional psychological mechanism. Fourth, the checklist-based behavioral findings were descriptive and exploratory, and cannot establish the causal effects or temporal ordering of specific coping strategies. Overall, the stronger coupling of threat and anxiety among international students further highlights the need for tailored support that accounts for the unique challenges faced by this population.

## 6 Contribution

### 6.1 Theoretical significance

This study evaluates whether perceived AI replacement threat, AI optimism, and employment anxiety can be represented as three related dimensions among domestic and international students studying at the same Chinese university. The findings support retaining this three-factor organization, while also showing that the distinction between threat and anxiety remains qualified, particularly among international students. The study further documents a stronger association between threat and anxiety in the international student sample. The small conditional association involving AI optimism is reported as an exploratory cross-sectional pattern rather than as evidence of a temporal mechanism.

### 6.2 Practical significance

The findings may inform local needs assessment for students facing AI-related employment uncertainty. Perceived employment threat was closely associated with anxiety, so institutions may consider assessing students’ concerns about skill obsolescence, employment competition, and career uncertainty when planning support. This study did not test whether skill training, career information, counseling, or particular coping strategies reduce anxiety. Any support program based on these findings therefore requires independent intervention evaluation before its effectiveness can be inferred. Whether changes in coping strategies lead to subsequent changes in anxiety or relief remains a question for longitudinal and intervention research.

## 7 Limitations and future directions

This study has several limitations. First, the cross-sectional design does not establish temporal ordering. The structural associations are compatible with several directional and reciprocal explanations. The equivalent fit of alternative threat-optimism specifications further limits directional interpretations. Longitudinal paneldiary, and intervention studies are needed to examine change over time. Second, the study relied on self-report measures. Shared response tendencies, recall, and social-desirability effects may influence the observed associations. Future studies could combine self-report data with repeated assessments, AI-use records, career services participation, or behavioral indicators where appropriate. Third, perceived AI replacement threat and employment anxiety were separately specified but remained closely related. The full-sample Fornell-Larcker comparison was borderline. Q11 includes affective worry wording, Q20 refers directly to AI replacement, and Q21 concerns general graduate employment. These item features may contribute to overlap between appraisal-related and emotional content. Cognitive interviewing, item revision, and independent replication are needed to clarify the empirical boundary between the two constructs. Fourth, only partial scalar invariance was obtained, and residual invariance was not established. Differences between domestic and international students may therefore reflect substantive variation, language interpretation, response style, employment destination, length of residence in China, or heterogeneity within the international student sample. The present design cannot distinguish these explanations. Future studies could use harmonized multilingual measures and larger country-specific samples. Fifth, the sample was recruited by convenience from one comprehensive university in China. The international-student group was diverse, but many country groups were small. The findings may not generalize to students in other institutional settings, labor markets, or national contexts. Multi-site studies with larger country-specific subsamples would provide a stronger basis for cross-cultural comparison. Sixth, Q25–Q28 were study-specific supplementary measures. Q27 assessed respondents’ overall perceived relief after they considered multiple selected coping strategies. It did not measure the effect of a particular strategy, and it cannot establish whether coping preceded or followed anxiety. These findings are descriptive and exploratory. Validated coping and support measures are needed before intervention effects are tested.

## Data Availability

The original contributions presented in the study are included in the article/Supplementary material, further inquiries can be directed to the corresponding author.
