# Supplementary material for: Generative AI-related employment threat, AI optimism, and employment anxiety among domestic and international students at a Chinese university
Source: Front Public Health. 2026 Oct 20;14:1927670. doi: 10.3389/fpubh.2026.1927670 (PMC13727444; doi:10.3389/fpubh.2026.1927670)
Supplement: Supplementary file 1 [file Supplementary_file_1.pdf]

Supplementary Material

1 Original Questionnaire (Bilingual)

The instrument was administered in two parallel versions: a Chinese version for domestic students and a bilingual version for international students. The two versions share an identical Likert item pool and only differ in (a) one demographic item (the international version includes “time in China” instead of “hometown”; this produces a one-position shift in numbering for items appearing after the demographic block), and (b) the open-ended final item, which appears as Q28 in the domestic version and Q29 in the international version. To preserve a unified scheme, the main manuscript uses the international version's numbering throughout.

Part 1. Demographic Characteristics and AI Familiarity (Q1-Q9)

Items Q1 through Q9 capture demographic characteristics, family economic context, and prior AI exposure. The numbering below follows the international student version. In the domestic student version, item Q4 (“How long have you been in China”) is replaced with “Place of origin” and the item is not used in any inferential analysis; subsequent items in the domestic version are numbered one position lower throughout.

No.	Chinese	English	Response options
Q1	您的性别	What is your gender?	1 = Male 2 = Female
Q2	您的年级	What is your grade level?	1 = Freshman 2 = Sophomore 3 = Junior 4 = Senior<br>5 = Master 6 = Doctoral 7 = Other
Q3	您的专业类别	What is your disciplinary category?	1 = STEM 2 = HSS 3 = EML 4 = Arts & PE<br>5 = Medicine 6 = Other
Q4 (international.)	您来自哪个国家/<br>你来中国多长时间了	Which country are you from /How long have you been in China?	Open/<br>1 = <6months 2 = 6–12months 3 = 1–2 years<br>4 = 2–3 years 5 = >3 years
Q4 (domestic.)	你的生源地是	What is your place of origin?	Open (Province + City)
Q5	您是否有过实习或兼职经历？	Have you had any internship or part-time work experience?	1 = Yes<br>2 = No
Q6	您家庭的经济压力状况	How would you describe your family’s economic pressure?	1 = None 2 = Mild<br>3 = Moderate 4 = Heavy
Q7	您对 AI 技术的了解程度	What is your level of understanding of AI technology?	1 = Very low 2 = Low 3 = Moderate<br>4 = High 5 = Very high
Q8	您是否使用过 AI 工具（如 DeepSeek、ChatGPT 等）	Have you used AI tools (e.g., DeepSeek, ChatGPT)?	1 = Frequently 2 = Occasionally<br>3 = Rarely 4 = Never

Part 2. Perceived AI Replacement Threat (Q10-Q13)

Items in Parts 2 through 4 are rated on a 5-point Likert scale (1 = strongly disagree, 2 = disagree, 3 = neutral, 4 = agree, 5 = strongly agree). The English wording reproduces the translation used in Table 2 of the main manuscript, with minor wording completion where the abbreviation in Table 2 omitted clarifying phrases.

No.	Chinese	English
Q10	我认为在未来 5-10 年内，AI 会替代大量现有的工作岗位	I believe that within the next 5–10 years, AI will replace a large number of existing jobs.
Q11	我担心自己所学的专业容易被 AI 替代	I worry that the major I am studying is easily replaceable by AI.
Q12	AI 技术会让我在求职时面临更激烈的竞争	AI technology will make me face fiercer competition when seeking a job.
Q13	即使我努力学习，AI 也可能让我的技能变得过时	Even if I study hard, AI may render my skills obsolete.

Part 3 & 4. AI Optimism (Q14-Q19)

Parts 3 (Attitudes toward AI, Q14-Q17) and 4 (Confidence in AI usage, Q18-Q19) of the original instrument were combined into a single “AI optimism” construct following the pilot study, as detailed in Section 2.2 of the main manuscript.

No.	Chinese	English
Q14	我认为 AI 是一种可以被我使用的工具，而不是威胁	I see AI as a tool I can use, not a threat.
Q15	我愿意主动学习 AI 工具来提升自己的竞争力	I am willing to actively learn AI tools to enhance my competitiveness.
Q16	我相信掌握 AI 技能的人在就业市场中更具优势	I believe that people who master AI skills have advantages in the job market.
Q17	AI 技术的发展会创造更多新的职业机会	The development of AI technology will create more new job opportunities.
Q18	我有信心学会使用常见的 AI 工具	I am confident that I can learn to use common AI tools.
Q19	我相信通过学习和使用 AI，我能增强自己的职业竞争力	I believe that by learning and using AI, I can enhance my career competitiveness.

Part 5. Employment Anxiety (Q20-Q23) and Emotional Tone (Q24)

No.	Chinese	English	Response options
Q20	想到 AI 可能替代我的工作，我会感到紧张不安	Thinking that AI may replace my job makes me feel tense and anxious.	5-point Likert
Q21	我担心自己毕业后找不到满意的工作	I worry that I will not find a satisfactory job after graduation.	5-point Likert
Q22	AI 技术的发展让我对未来职业道路感到迷茫	The development of AI technology makes me feel confused about my future career path.	5-point Likert
Q23	我对自己能否在 AI 时代保持竞争力缺乏信心	I lack confidence in whether I can remain competitive in the AI era.	5-point Likert
Q24	您接触到的“AI 与就业”相关信息，整体情绪倾向是	What is the emotional tone of the encountered information related to AI and employment?	1 = Very negative<br>2 = Negative<br>3 = Neutral<br>4 = Positive<br>5 = Very positive

Q20 to Q23 measure career anxiety. Q24 captures the perceived emotional tone of encountered information related to AI and employment, and is used as a covariate in robustness checks rather than as a construct indicator.

Part 6. Multi-Response Items and Open-Ended Question (Q25-Q29)

Q25. Information channels

您主要从哪些渠道了解？（多选）

Which channels do you mainly use to learn about AI and employment? (multi-select)

Option	Chinese	English
A	新闻报道	News reports
B	学术论文/研究报告	Academic papers / research reports
C	微信公众号	WeChat public accounts
D	APP 推送（抖音/B 站/知乎/TikTok/小红书 等）	APP notifications (Douyin / Bilibili / Zhihu / TikTok / RedNote, etc.)
E	与同学/老师讨论	Discussions with classmates / teachers
F	学校课程/讲座	School courses / lectures

Q26. Coping behaviors

您采取了哪些应对措施？（多选）

Which coping behaviors have you adopted? (multi-select)

Option	Chinese	English
A	主动学习 AI 相关技能	Active AI-skill learning
B	与同学/老师/家人倾诉	Talking to classmates / teachers / family
C	增加实习/项目经历	Increasing internship / project experience
D	运动锻炼	Exercise
E	听音乐/看电影	Listening to music / watching movies (entertainment)
F	暂时不去想（回避）	Temporarily not thinking about it (avoidance)
G	尝试看到 AI 带来的新机会（认知重构）	Trying to see new opportunities from AI (cognitive reappraisal)
H	其他	Other

Q27. Self-reported perceived relief after coping

您认为上述方式缓解焦虑的整体效果如何？

How effective do you think these coping methods are in alleviating your anxiety?

Response options: 1 = Very ineffective 2 = Somewhat ineffective

3 = Neutral 4 = Somewhat effective 5 = Very effective

Q28. Desired institutional support (multi-select)

您希望学校提供哪些支持？（多选）

What kinds of support would you like the school to provide? (multi-select)

Option	Chinese	English
A	开设 AI 与职业发展融合课程	AI–career integration courses
B	提供个性化职业咨询	Personalized career counseling
C	组织 AI 技能工作坊	AI skill workshops
D	增加实习内推机会	Internship referral opportunities
E	定期开展就业心理普查	Regular employment-psychology screening
F	其他	Other

Q29. Open-ended question

请用一两句话描述您对 AI 与未来就业最真实的感受：

Please describe in one or two sentences your most honest feelings about AI and your future career:

Open response. Free-text responses were not analyzed quantitatively in the present study and are not reported in the main manuscript.

Table S1. National and regional distribution of international students.

Category	Group	N	Percentage of international sample (%)
By region	Asia	337	67.4
	Africa	124	24.8
	Europe	22	4.4
	South America	7	1.4
	North America	7	1.4
	Oceania	3	0.6
	Total	500	100.0
Top 4 countries	Pakistan	89	17.8
	Turkmenistan	40	8.0
	Cambodia	38	7.6
	Indonesia	37	7.4
Others	Remaining 74 countries/regions	296	59.2
Total	—	500	100.0

2 Exploratory factor analysis

Parallel analysis suggested three factors in both groups. Principal-axis factoring with Promax rotation was then used. Full-sample KMO was 0.89; Bartlett's $\chi^2(78) = 12{,}515.87$, $p < 0.001$. EFA and CFA used the same main sample and therefore do not constitute independent validation. Besides, dashes denote coefficients suppressed in the displayed factor-loading output; they are not zero estimates.

Table S2a. Domestic EFA loadings.

Item	Threat	Optimism	Anxiety
Q11	0.720	—	—
Q12	0.764	—	—
Q13	0.723	—	—
Q14	—	0.698	—
Q15	—	0.884	—
Q16	—	0.848	—
Q17	—	0.689	—
Q18	—	0.760	—
Q19	—	0.828	—
Q20	—	—	0.691
Q21	—	—	0.754
Q22	—	—	0.894
Q23	—	—	0.873

Table S2a. International EFA loadings.

Item	Threat	Optimism	Anxiety
Q11	0.543	—	0.369
Q12	0.547	—	—
Q13	0.474	—	—
Q14	—	0.738	—
Q15	—	0.852	—
Q16	—	0.814	—
Q17	—	0.634	—
Q18	—	0.769	—
Q19	—	0.821	—
Q20	—	—	0.706
Q21	—	—	0.844
Q22	—	—	0.948
Q23	—	—	0.847

Table S2a. Full sample EFA loadings.

Item	Threat	Optimism	Anxiety
Q11	0.693	—	—
Q12	0.744	—	—
Q13	0.689	—	—
Q14	—	0.715	—
Q15	—	0.873	—
Q16	—	0.832	—
Q17	—	0.665	—
Q18	—	0.767	—
Q19	—	0.829	—
Q20	—	—	0.676
Q21	—	—	0.797
Q22	—	—	0.913
Q23	—	—	0.871

Table S2b. EFA variance and factor correlations.

Sample	Item variance explained	Threat–optimism	Threat–anxiety	Optimism–anxiety
Domestic	62.3%	0.047	0.678	−0.046
International	58.6%	0.272	0.566	−0.085
Full sample	61.5%	—	—	—

3 Confirmatory measurement analyses and sensitivity checks

Table S3a. Three-factor CFA fit.

Model / sample	Scaled χ^2 (df)	CFI	TLI	RMSEA	SRMR
Full sample	368.609 (61)	0.966	0.957	0.066	0.064
Domestic	335.290 (61)	0.954	0.941	0.077	0.074
International	162.342 (61)	0.969	0.961	0.062	0.063

Table S3b. Standardized full-sample loadings.

Item	Construct	Loading
Q11		0.748
Q12	Threat	0.746
Q13		0.737
Q14		0.695
Q15		0.894
Q16		0.859
Q17	Optimism	0.657
Q18		0.721
Q19		0.789
Q20		0.800
Q21		0.759
Q22	Anxiety	0.890
Q23		0.873

Table S3c. Full-sample reliability and convergent validity.

Construct	Cronbach α	CR	AVE	√AVE
Threat	0.787	0.788	0.553	0.744
Optimism	0.900	0.898	0.599	0.774
Anxiety	0.897	0.900	0.692	0.832

Table S3d. HTMT by sample.

Construct pair	Full sample	Domestic	International
Threat / optimism	0.094	0.120	0.118
Threat / anxiety	0.752	0.707	0.813
Optimism / anxiety	0.064	0.078	0.051

Table S3e. Item and estimator sensitivity analyses.

Model / sample	Scaled χ^2 (df)	CFI	TLI	RMSEA	SRMR
Retain Q10, 14 items	558.959 (73)	0.950	0.937	0.075	0.081
Remove Q11 and Q20	256.752 (40)	0.970	0.959	0.069	0.059
Remove Q20 only	314.097 (50)	0.967	0.957	0.067	0.064
No Q18-Q19 residual covariance	563.034 (62)	0.943	0.929	0.084	0.066
Ordinal WLSMV	1592.874 (61)	0.961	0.951	0.126	0.076

After removing Q11 and Q20, the correlation between threat and anxiety was 0.700; after removing Q20 alone, it was 0.723. With Q10 retained, threat CR was 0.783 and AVE was 0.480. The corresponding threat–anxiety correlations were 0.679 [0.610, 0.739] for domestic students and 0.793 [0.729, 0.854] for international students, with $\Delta r = 0.114$ [0.024, 0.205], $p = 0.012$. These analyses address item dependence but do not eliminate concerns about wording or discriminant validity.

When domestic subsamples of 500 were analyzed, three factors were retained in 73% of replications, two in 26%, and four in 1%. This result indicates some sensitivity of factor retention to resampling. It does not exclude sample-size or sampling effects.

Table S3f. Ten largest modification indices in the retained model.

Candidate parameter	MI	EPC	Standardized EPC
Threat loading on Q14	116.933	−0.243	−0.227
Anxiety loading on Q14	106.457	−0.218	−0.204
Optimism loading on Q12	63.970	0.287	0.174
Optimism loading on Q11	61.344	−0.306	−0.170
Threat loading on Q16	59.257	0.135	0.128
Anxiety loading on Q16	47.682	0.114	0.109
Optimism loading on Q21	47.518	0.232	0.127
Q11-Q20 residual covariance	47.474	0.090	0.214
Threat loading on Q20	34.263	0.241	0.193
Q14-Q17 residual covariance	29.139	0.064	0.150

Table S3g. Largest standardized residual diagnostic entries.

Item pair	Diagnostic value
Q14 / Q11	9.126
Q21 / Q16	8.346
Q16 / Q12	8.201
Q22 / Q14	7.779
Q23 / Q14	7.619

Item pair	Diagnostic value
Q20 / Q14	6.568
Q14 / Q13	6.281
Q21 / Q15	5.867
Q17 / Q11	5.743
Q20 / Q11	5.251

Table S3h. Merged-factor sensitivity check.

Model / sample	Scaled χ^2 (df)	CFI	TLI	RMSEA	SRMR
Domestic merged threat/anxiety	634.563 (63)	0.902	0.879	0.110	0.089
International merged threat/anxiety	253.685 (63)	0.942	0.928	0.084	0.084

4 Measurement invariance and qualified group comparisons

Table S4a. Measurement invariance.

Model	χ^2 (df)	CFI	TLI	RMSEA	SRMR	ΔCFI
Configural	675.932 (122)	0.956	0.943	0.075	0.066	-0.008
Metric	709.202 (132)	0.954	0.945	0.074	0.069	-0.002
Scalar	856.842 (142)	0.943	0.937	0.079	0.072	-0.011
Partial scalar	734.955 (141)	0.953	0.947	0.073	0.069	-0.001

Table S4b. Likelihood information criteria.

Model	AIC	BIC
Configural	43587.352	44049.571
Metric	43600.622	44009.094
Scalar	43728.262	44082.988
Partial scalar	43608.375	43968.475

The largest intercept score test concerned Q21, $\chi^2(1) = 117.724$, followed by Q20 (20.862) and Q22 (14.791). Other nonzero score tests remained, including Q20 loading equality (54.699). Partial scalar invariance is therefore an approximate basis for interpretation; freeing Q21 does not imply that every remaining parameter is invariant. Residual invariance was not established for this revised model.

Table S4c. Threat–anxiety correlations across invariance specifications.

Model	Domestic r [95% CI]	International r [95% CI]	Δr [95% CI]	p for Δr
Configural	0.697 [0.629, 0.758]	0.823 [0.763, 0.878]	0.126 [0.040, 0.214]	0.005
Metric	0.698 [0.631, 0.760]	0.819 [0.759, 0.877]	0.121 [0.035, 0.212]	0.007
Scalar	0.698 [0.631, 0.760]	0.820 [0.760, 0.879]	0.122 [0.036, 0.215]	0.007
Partial scalar	0.698 [0.630, 0.759]	0.819 [0.760, 0.877]	0.121 [0.036, 0.213]	0.007

Note. Δr = international minus domestic. Intervals are percentile bootstrap intervals. Equality of structural regression paths is not inferred from these correlation comparisons.

Table S4d. Latent mean differences under partial scalar invariance.

Construct	ΔM	SE	95% CI	p	Standardized ΔM
Threat	-0.241	0.050	[-0.341, -0.144]	< 0.001	-0.294
Optimism	0.051	0.035	[-0.017, 0.120]	0.140	0.081
Anxiety	-0.358	0.047	[-0.448, -0.258]	< 0.001	-0.414

Note. International minus domestic; domestic latent means are fixed at zero. Standardization uses domestic latent SDs of 0.820, 0.636, and 0.865 for threat, optimism, and anxiety. Intervals are percentile bootstrap intervals. Under full scalar invariance the anxiety difference was −0.423 [−0.514, −0.324]; threat and optimism differences were unchanged at the displayed precision.

5 Structural specifications and covariate adjustment

The model was further examined in two robustness analyses. First, after adding AI familiarity and usage frequency as covariates, the association between threat and optimism remained positive and significant (β = 0.121, *p* = 0.004; primary model: β = 0.133), and the indirect effect remained negative and significant (β = -0.017, 95% CI [-0.035, -0.005], *p* = 0.020). AI familiarity and usage frequency were both positively associated with optimism (β = 0.187 and 0.166), and usage frequency was also positively associated with threat (β = 0.126). Besides, a PROCESS Model 4 analysis using observed composite scores yielded the same pattern: (a×b = -0.0094, 95% BootCI [-0.0181, -0.0018]). The observed-score model explained 41.4% of the variance in anxiety.

Table S5a. Primary structural estimates.

Term	Path	b	β	SE	p	95% CI
Path a	Threat → Optimism	0.099	0.133	0.032	0.002	(0.036, 0.164)
Path b	Optimism → Anxiety	−0.205	-0.152	0.033	< 0.001	(−0.266, −0.139)
Direct (c′)	Threat → Anxiety	0.765	0.764	0.037	< 0.001	(0.694, 0.837)
Indirect effect (a × b)	Threat → Optimism → Anxiety	−0.020	-0.020	0.008	0.012	(-0.039, -0.007)
Total effect	Threat → Anxiety	0.745	0.744	0.035	< 0.001	(0.678,0.814)

Table S5b. Equivalent directional specifications.

Threat–optimism specification	Scaled χ^2 (df)	CFI	TLI	RMSEA	SRMR
Optimism regressed on threat	368.609 (61)	0.966	0.957	0.066	0.064
Threat regressed on optimism	368.609 (61)	0.966	0.957	0.066	0.064
Threat and optimism covary	368.609 (61)	0.966	0.957	0.066	0.064

Note. All specifications retain their associations with anxiety. MLR fit: RMSEA 90% CI [0.059, 0.072], AIC = 43983.10, BIC = 44144.34 for all three. Equivalent fit does not identify causal direction.

Table S5c. Structural model adjusted for AI familiarity and use frequency.

Outcome / predictor	b	SE	β	p	95% CI for b
Threat / familiarity	-0.067	0.033	-0.066	0.041	[-0.132, −0.003]
Threat / use frequency	0.200	0.042	0.126	<0 .001	[0.119, 0.286]
Optimism / threat	0.090	0.031	0.121	0.004	[0.029, 0.151]
Optimism / familiarity	0.142	0.023	0.187	< 0.001	[0.096, 0.186]
Optimism / use frequency	0.196	0.033	0.166	< 0.001	[0.129, 0.262]
Anxiety / optimism	-0.190	0.034	-0.142	< 0.001	[-0.255, −0.122]
Anxiety / threat	0.756	0.037	0.755	< 0.001	[0.685, 0.828]

Outcome / predictor	b	SE	β	p	95% CI for b
Anxiety / familiarity	-0.089	0.023	-0.087	< 0.001	[-0.134, -0.045]
Anxiety / use frequency	0.072	0.032	0.046	0.023	[0.013, 0.137]
Indirect association	-0.017	0.007	-0.017	0.020	[-0.035, -0.005]

Note. ML with 5,000 bootstrap resamples; bias-corrected intervals. Covariate coefficients refer to the analysis-coded familiarity and frequency variables. The adjustment addresses measured AI exposure and does not exclude other common causes.

6 Observed-score indirect association

PROCESS Model 4 used centered threat and optimism composites and employment anxiety as the outcome (N = 1,595). The optimism equation had $R^2 = 0.0066$, $F(1, 1593) = 10.643$, $p = 0.0011$. The anxiety equation had $R^2 = 0.4139$, $F(2, 1592) = 562.146$, $p < 0.0001$.

Table S6. PROCESS Model 4 estimates.

Term	b	SE	t	p	95% CI
Threat / optimism equation	0.0651	0.0200	3.262	0.0011	[0.0260, 0.1043]
Threat / anxiety equation	0.6737	0.0202	33.393	< 0.0001	[0.6342, 0.7133]
Optimism / anxiety equation	−0.1449	0.0252	−5.741	< 0.0001	[−0.1945, −0.0954]
Indirect association	−0.0094	0.0041	—	—	[−0.0181, −0.0018]

Note. The indirect-association SE and interval use 5,000 bootstrap resamples. Other intervals are coefficient confidence intervals. Model 4 and the interaction model in Table S7 estimate different conditional relationships; differences between their optimism coefficients should not be attributed solely to centering.

7 Observed-score indirect association

A separate PROCESS Model 1 analysis showed that the interaction between threat and optimism was statistically significant but minimal (b = -0.0518, p = 0.0221, ΔR^2 = 0.0019). The conditional association between threat and anxiety decreased modestly as optimism increased, from b = 0.7199, 95% CI [0.6640, 0.7758] at the 16th percentile of optimism to b = 0.6767, 95% CI [0.6371, 0.7163] at the median and b = 0.6421, 95% CI [0.5942, 0.6900] at the 84th percentile.

Table S7a. Moderation coefficients.

Term	b	SE	t	p	95% CI
Intercept	0.8633	0.2947	2.930	0.0034	[0.2853, 1.4413]
Threat	0.8840	0.0940	9.408	< 0.0001	[0.6997, 1.0684]
Optimism	0.0083	0.0715	0.116	0.9075	[−0.1319, 0.1485]
Threat × optimism	−0.0518	0.0226	−2.291	0.0221	[−0.0962, −0.0075]

Note. The threat and optimism coefficients are conditional on the other predictor being zero in this uncentered interaction model. They should not be interpreted as unconditional associations.

Table S7b. Conditional threat–anxiety associations.

Optimism percentile	Score	b	SE	t	95% CI
16th	3.1667	0.7199	0.0285	25.271	[0.6640, 0.7758]
50th	4.0000	0.6767	0.0202	33.516	[0.6371, 0.7163]
84th	4.6667	0.6421	0.0244	26.293	[0.5942, 0.6900]

Note. All three p values are < 0.0001. The interaction accounts for an additional 0.19% of variance and does not establish an intervention effect.

8 Multi-response module

Multiple responses were permitted. Each row compares endorsement versus non-endorsement between groups. These checklist results are exploratory.

Table S8. Descriptive statistics of items with multiple responses.

Item	Choices	Total	Domestic	International	χ^2	*p*
Q25 Information channel	APP notifications	65.6%	75.5%	44.0%	151.26	< 0.001
	news reports	61.3%	68.5%	45.4%	77.13	< 0.001
	talking with classmates / teachers	52.0%	50.3%	55.2%	3.27	0.070
	academic papers / reports	51.0%	48.4%	57.0%	10.16	0.001
	WeChat public accounts	47.0%	53.6%	31.6%	66.79	< 0.001
	school courses / lectures	38.6%	35.6%	45.0%	12.76	< 0.001
Q26 Coping behavior	active AI-skill learning	76.9%	80.7%	68.6%	28.46	< 0.001
	cognitive reappraisal	53.2%	57.7%	43.4%	28.26	< 0.001
	increasing internship experience	40.1%	42.8%	34.2%	10.64	0.001
	talking to others	32.9%	29.6%	40.0%	16.87	< 0.001
	exercise	32.3%	31.0%	35.2%	2.82	0.093
	entertainment	30.3%	32.9%	24.4%	11.70	0.001
	avoidance	14.6%	16.7%	10.0%	12.40	< 0.001
	other	7.5%	5.0%	12.8%	30.07	< 0.001
Q28 Desired support	courses integrated AI and career	65.3%	66.6%	62.6%	2.39	0.122
	internship referrals	63.3%	66.9%	55.2%	20.36	< 0.001
	AI skill workshops	57.4%	56.1%	60.2%	2.39	0.122
	personalized career counseling	47.3%	50.5%	40.2%	14.62	< 0.001
	regular employment-psychology screening	25.8%	26.4%	24.6%	0.58	0.448
	other	8.1%	5.4%	14.0%	34.25	< 0.001

9 Coping endorsement analyses

Q27 is a single study-specific report of overall perceived relief after multiple selected coping methods. It does not assess the effect of an individual strategy. The analyses are cross-sectional and exploratory.

Table S9. Group differences in worry, AI optimism, and perceived coping relief as a function of coping strategy endorsement.

Coping behavior	Outcome variable	Welch's t	*df*	*p*	Hedges'g [95% CI]	Holm-corrected inference
Temporary avoidance	Employment anxiety	8.462	306.90	< 0.001	0.625 [0.484, 0.765]	Significant
	AI optimism	1.996	319.60	0.047	−0.140 [−0.279, −0.001]	Not significant
	Self-reported perceived relief after coping (Q27)	4.099	312.28	< 0.001	−0.296 [−0.435, −0.156]	Significant
Cognitive reappraisal	Employment anxiety	1.421	1554.00	0.156	−0.071 [−0.027, 0.169]	Not significant
	AI optimism	10.540	1484.83	< 0.001	0.534 [0.433, 0.634]	Significant
	Self-reported perceived relief after coping (Q27)	8.561	1511.52	< 0.001	0.432 [0.333, 0.532]	Significant
